# Management and outcomes in critically ill nonagenarian versus octogenarian patients

**DOI:** 10.1186/s12877-021-02476-4

**Published:** 2021-10-19

**Authors:** Raphael Romano Bruno, Bernhard Wernly, Malte Kelm, Ariane Boumendil, Alessandro Morandi, Finn H. Andersen, Antonio Artigas, Stefano Finazzi, Maurizio Cecconi, Steffen Christensen, Loredana Faraldi, Michael Lichtenauer, Johanna M. Muessig, Brian Marsh, Rui Moreno, Sandra Oeyen, Christina Agvald Öhman, Bernardo Bollen Pinto, Ivo W. Soliman, Wojciech Szczeklik, Andreas Valentin, Ximena Watson, Susannah Leaver, Carole Boulanger, Sten Walther, Joerg C. Schefold, Michael Joannidis, Yuriy Nalapko, Muhammed Elhadi, Jesper Fjølner, Tilemachos Zafeiridis, Dylan W. De Lange, Bertrand Guidet, Hans Flaatten, Christian Jung, on behalf of the VIP2 study group, Michael Joannidis, Philipp Eller, Raimund Helbok, René Schmutz, Joke Nollet, Nikolaas de Neve, Pieter De Buysscher, Sandra Oeyen, Walter Swinnen, Marijana Mikačić, Anders Bastiansen, Andreas Husted, Bård E. S. Dahle, Christine Cramer, Christoffer Sølling, Dorthe Ørsnes, Jakob Edelberg Thomsen, Jonas Juul Pedersen, Mathilde Hummelmose Enevoldsen, Thomas Elkmann, Agnieszka Kubisz-Pudelko, Alan Pope, Amy Collins, Ashok S. Raj, Carole Boulanger, Christian Frey, Ciaran Hart, Clare Bolger, Dominic Spray, Georgina Randell, Helder Filipe, Ingeborg D. Welters, Irina Grecu, Jane Evans, Jason Cupitt, Jenny Lord, Jeremy Henning, Joanne Jones, Jonathan Ball, Julie North, Kiran Salaunkey, Laura Ortiz-Ruiz De Gordoa, Louise Bell, Madhu Balasubramaniam, Marcela Vizcaychipi, Maria Faulkner, Mc Donald Mupudzi, Megan Lea-Hagerty, Michael Reay, Michael Spivey, Nicholas Love, Nick Spittle Nick Spittle, Nigel White, Patricia Williams, Patrick Morgan, Phillipa Wakefield, Rachel Savine, Reni Jacob, Richard Innes, Ritoo Kapoor, Sally Humphreys, Steve Rose, Susan Dowling, Susannah Leaver, Tarkeshwari Mane, Tom Lawton, Vongayi Ogbeide, Waqas Khaliq, Yolanda Baird, Antoine Romen, Arnaud Galbois, Bertrand Guidet, Christophe Vinsonneau, Cyril Charron, Didier Thevenin, Emmanuel Guerot, Guillaume Besch, Guillaume Savary, Hervé Mentec, Jean-Luc Chagnon, Jean-Philippe Rigaud, Jean-Pierre Quenot, Jeremy Castanera, Jérémy Rosman, Julien Maizel, Kelly Tiercelet, Lucie Vettoretti, Maud Mousset Hovaere, Messika Messika, Michel Djibré, Nathalie Rolin, Philippe Burtin, Pierre Garcon, Saad Nseir, Xavier Valette, Christian Rabe, Eberhard Barth, Henning Ebelt, Kristina Fuest, Marcus Franz, Michael Horacek, Michael Schuster, Patrick Meybohm, Raphael Romano Bruno, Sebastian Allgäuer, Simon Dubler, Stefan J. Schaller, Stefan Schering, Stephan Steiner, Thorben Dieck, Tim Rahmel, Tobias Graf, Anastasia Koutsikou, Aristeidis Vakalos, Bogdan Raitsiou, Elli Niki Flioni, Evangelia Neou, Fotios Tsimpoukas, Georgios Papathanakos, Giorgos Marinakis, Ioannis Koutsodimitropoulos, Kounougeri Aikaterini, Nikoletta Rovina, Stylliani Kourelea, Polychronis Tasioudis, Vasiiios Zidianakis, Vryza Konstantinia, Zoi Aidoni, Brian Marsh, Catherine Motherway, Chris Read, Ignacio Martin-Loeches, Andrea Neville Cracchiolo, Aristide Morigi, Italo Calamai, Stefania Brusa, Ahmed Elhadi, Ahmed Tarek, Ala Khaled, Hazem Ahmed, Wesal Ali Belkhair, Alexander D. Cornet, Diederik Gommers, Dylan W. De Lange, Eva van Boven, Jasper Haringman, Lenneke Haas, Lettie van den Berg, Oscar Hoiting, Peter de Jager, Rik T. Gerritsen, Tom Dormans, Willem Dieperink, Alena Breidablik Alena Breidablik, Anita Slapgard, Anne-Karin Rime, Bente Jannestad, Britt Sjøbøe, Eva Rice, Finn H. Andersen, Hans Frank Strietzel, Jan Peter Jensen, Jørund Langørgen, Kirsti Tøien, Kristian Strand, Michael Hahn, Pål Klepstad, Aleksandra Biernacka, Anna Kluzik, Bartosz Kudlinski, Dariusz Maciejewski, Dorota Studzińska, Hubert Hymczak, Jan Stefaniak, Joanna Solek-Pastuszka, Joanna Zorska, Katarzyna Cwyl, Lukasz J. Krzych, Maciej Zukowski, Małgorzata Lipińska-Gediga, Marek Pietruszko, Mariusz Piechota, Marta Serwa, Miroslaw Czuczwar, Mirosław Ziętkiewicz, Natalia Kozera, Paweł Nasiłowski, Paweł Sendur, Paweł Zatorski, Piotr Galkin, Ryszard Gawda, Urszula Kościuczuk, Waldemar Cyrankiewicz, Wojciech Gola, Alexandre Fernandes Pinto, Ana Margarida Fernandes, Ana Rita Santos, Cristina Sousa, Inês Barros, Isabel Amorim Ferreira, Jacobo Bacariza Blanco, João Teles Carvalho, Jose Maia, Nuno Candeias, Nuno Catorze, Vladislav Belskiy, Africa Lores, Angela Prado Mira, Catia Cilloniz, David Perez-Torres, Emilio Maseda, Enver Rodriguez, Estefania Prol-Silva, Gaspar Eixarch, Gemma Gomà, Gerardo Aguilar, Gonzalo Navarro Velasco, Marián Irazábal Jaimes, Mercedes Ibarz Villamayor, Noemí Llamas Fernández, Patricia Jimeno Cubero, Sonia López-Cuenca, Teresa Tomasa, Anders Sjöqvist, Camilla Brorsson, Fredrik Schiöler, Henrik Westberg, Jessica Nauska, Joakim Sivik, Johan Berkius, Karin Kleiven Thiringer, Lina De Geer, Sten Walther, Filippo Boroli, Joerg C. Schefold, Leila Hergafi, Philippe Eckert, Ismail Yıldız, Ihor Yovenko, Yuriy Nalapko, Richard Pugh

**Affiliations:** 1 Department of Cardiology, Pulmonary Diseases, and Vascular Medicine, Medical Faculty, Heinrich Heine University of Duesseldorf, Moorenstraße 5, 40225 Duesseldorf, Germany; 2 Department of Anaesthesiology, Perioperative Medicine and Intensive Care Medicine, Paracelsus Medical University, Salzburg, Austria; 3 Division of Cardiology, Department of Medicine, Karolinska Institutet, Karolinska University Hospital, Stockholm, Sweden; 4 Cardiovascular Research Institute Düsseldorf (CARID), Duesseldorf, Germany; 5 Service de Réanimation Médicale, Publique-Hôpital de Paris, Hôpital Saint-Antoine, F-75012 Paris, France; 6 Department of Rehabilitation Hospital Ancelle di Cremona, Cremona, Italy; 7 Geriatric Research Group, Brescia, Italy; 8 Department Of Anaesthesia and Intensive Care, Ålesund Hospital, Ålesund, Norway; 9 NTNU, Dep of Circulation and Medical Imaging, Trondheim, Norway; 10 Department of Intensive Care Medicine, CIBERes Corporacion Sanitaria Universitaria Parc Tauli, Barcelona, Spain; 11 Istituto di Ricerche Farmacologiche Mario Negri IRCCS, Ranica, BG Italy; 12 Department of Anaesthesia, IRCCS Instituto Clínico Humanitas, Humanitas University, Milan, Italy; 13 Department of Anaesthesia and Intensive Care Medicine, Aarhus University Hospital, Aarhus, Denmark; 14 Grande Ospedale Metropolitano Niguarda, Milan, Italy; 15 Department of Cardiology, Paracelsus Medical University, Salzburg, Austria; 16 Mater Misericordiae University Hospital, Dublin, Ireland; 17 Unidade de Cuidados Intensivos Neurocríticos e Trauma, Faculdade de Ciências Médicas de Lisboa, Hospital de São José, Centro Hospitalar Universitário de Lisboa Central, Nova Médical School, Lisbon, Portugal; 18 Department of Intensive Care, 1K12IC Ghent University Hospital, Ghent, Belgium; 19 Karolinska University Hospital, Solna, Sweden; 20 Department of Acute Medicine, Geneva University Hospitals, Geneva, Switzerland; 21 Department of Intensive Care Medicine, University Medical Center, University Utrecht, Utrecht, The Netherlands; 22 Intensive Care and Perioperative Medicine Division, Jagiellonian University Medical College, Kraków, Poland; 23 Kardinal Schwarzenberg Hospital, Schwarzach, Austria; 24 St George’s University Hospital, London, UK; 25 Research Lead Critical Care Directorate St George’s Hospital, London, UK; 26 NAHP Committee ESICM, Intensive Care Unit, Royal Devon & Exeter NHS Foundation Trust, Exeter, UK; 27 Linkoping University Hospital, Linkoping, Sweden; 28 Department of Intensive Care Medicine, Inselspital, Universitätsspital, University of Bern, Bern, Switzerland; 29 Division of Intensive Care and Emergency Medicine, Department of Internal Medicine, Medical University Innsbruck, Innsbruck, Austria; 30 European Wellness International, ICU, Luhansk, Ukraine; 31 Alkhums Hospital, ICU, Tripoli, Libya; 32 Department of Intensive Care, Aarhus University Hospital, Aarhus, Denmark; 33 Intensive Care Unit General Hospital of Larissa, Larissa, Greece; 34 Sorbonne Universités, UPMC Univ Paris 06, UMR_S 1136, Institut Pierre Louis d’Epidémiologie et de Santé Publique, F-75013 Paris, France; 35 INSERM, UMR_S 1136, Institut Pierre Louis d’Epidémiologie et de Santé Publique, F-75013 Paris, France; 36 Department of Clinical Medicine, University of Bergen, Bergen, Norway; 37 Department of Anaestesia and Intensive Care, Haukeland University Hospital, Bergen, Norway

**Keywords:** Octogenarians, Nonagenarians, Frailty, Intensive care medicine, Outcome

## Abstract

**Background:**

Intensive care unit (ICU) patients age 90 years or older represent a growing subgroup and place a huge financial burden on health care resources despite the benefit being unclear. This leads to ethical problems. The present investigation assessed the differences in outcome between nonagenarian and octogenarian ICU patients.

**Methods:**

We included 7900 acutely admitted older critically ill patients from two large, multinational studies. The primary outcome was 30-day-mortality, and the secondary outcome was ICU-mortality. Baseline characteristics consisted of frailty assessed by the Clinical Frailty Scale (CFS), ICU-management, and outcomes were compared between octogenarian (80–89.9 years) and nonagenarian (> 90 years) patients. We used multilevel logistic regression to evaluate differences between octogenarians and nonagenarians.

**Results:**

The nonagenarians were 10% of the entire cohort. They experienced a higher percentage of frailty (58% vs 42%; *p* < 0.001), but lower SOFA scores at admission (6 + 5 vs. 7 + 6; *p* < 0.001). ICU-management strategies were different. Octogenarians required higher rates of organ support and nonagenarians received higher rates of life-sustaining treatment limitations (40% vs. 33%; *p* < 0.001). ICU mortality was comparable (27% vs. 27%; *p* = 0.973) but a higher 30-day-mortality (45% vs. 40%; *p* = 0.029) was seen in the nonagenarians. After multivariable adjustment nonagenarians had no significantly increased risk for 30-day-mortality (aOR 1.25 (95% CI 0.90–1.74; *p* = 0.19)).

**Conclusion:**

After adjustment for confounders, nonagenarians demonstrated no higher 30-day mortality than octogenarian patients. In this study, being age 90 years or more is no particular risk factor for an adverse outcome. This should be considered– together with illness severity and pre-existing functional capacity - to effectively guide triage decisions.

**Trial registration:**

NCT03134807 and NCT03370692.

## Introduction

The proportion of older patients has increased significantly over time. In 2030, there will be more than 30 million people over the age of 90 (nonagenarians) in 35 industrialised countries [1]. Consequently, health care providers nowadays perform medical procedures on very old patients (from surgery to oncological therapies), which were previously considered unfeasible because of age or age-related deterioration in physical and mental performance [2]. Similarly, the rate of older patients (> 80 years) in intensive care units (ICU) is increasing [3–6]. Today, older patients utilise a disproportionate amount of health care resources compared to their relative proportion of the total population [3, 7].

In particular, the extent to which “old age” per se is a risk factor and the extent to which different groups of old patients differ from one another regarding the prognosis is the subject of continuing debate. Older patients suffer worse outcomes than younger patients undergoing intensive care [8, 9], but some studies failed to establish age as an independent predictor of mortality in older ICU patients [10, 11]. However, most prognostic studies demonstrated an almost linear relationship between chronological age and mortality after the age of 40 [12]. In this respect, patients ageing 80 years and more represent a particular challenge to intensive care medicine [13, 14]. Still, there are no large studies that further differentiate this group of very old ICU patients and it is unclear if being a nonagenarian is a risk factor for adverse outcomes. We hypothesize that critically ill nonagenarians have an elevated 30-day mortality compared to octogenarians. To address this hypothesis, we performed a retrospective cohort study comprised of two large, multinational prospective observational cohorts [13–15]. This post-hoc analysis combined data from the VIP-1 and VIP-2 studies to compare octo- and nonagenarians regarding 30-day mortality (primary outcome) and ICU mortality (secondary outcome), the distribution of risk factors, and the intensive care management [13–15].

## Methods

### Study subjects

The very old intensive care patients (VIP) studies, VIP1 and VIP2, were prospective, multi-centre studies, registered on ClinicalTrials.gov (ID: NTC03134807, NCT03370692). Both studies included very old intensive care patients (VIPs), defined as patients admitted to an ICU and aged 80 years or older. The main results from these studies have been published previously [13, 14, 16, 17]. In summary, for both studies, each participating ICU could include either consecutive patients admitted over a six-month period or the first 20 consecutive patients fulfilling the inclusion criteria (all patients aged 80 years or older). The data collection for VIP1 took place between October 2016 and February 2017 and between May 2018 to May 2019 for VIP2. Both studies used similar inclusion criteria as described elsewhere [13]. Informed consent was obtained from study participants. Local ethical committees might have waived the need of informed consent.

In this post-hoc analysis of these two studies, all patients admitted acutely (non-electively) with complete data on age, gender, clinical frailty score (CFS), sequential organ failure assessment (SOFA) score, and ICU mortality were included. For this study, the elective patients included in VIP1 were excluded as their outcomes differ significantly compared with those admitted acutely, as previously shown [18]. The primary endpoint of this study was ICU-mortality, and the secondary endpoint was 30-day-mortality.

### Scales, scores, and limitations in life-sustaining therapy

The SOFA score was recorded on admission; it could be calculated manually or using an online calculator. Frailty was assessed by the clinical frailty scale (CFS). The CSF distinguishes nine classes of frailty from very fit (CFS 1) to terminally ill (CFS 9). The respective visual and simple description for this assessment tool was used with permission [19–21].

The Katz Activities of Daily Living (Katz ADL) scale is a widely used graded instrument to assess disability in chronically ill or older patients. It evaluates six primary and psychosocial functions: bathing, dressing, going to the toilet, transferring, feeding, and continence. The patient receives 1 point for every independent and 0 for every dependent activity (6 = independent patient, 0 = very dependent patient). For the patients in the VIP2 trial, disability was defined by Katz ADL score ≤ 4.

For cognitive decline, VIP2 utilised the Short form of Informant Questionnaire on Cognitive Decline in the Elderly (IQCODE). IQCODE is a questionnaire, completed by carers, with 16 questions about cognitive decline over the past 10 years. For each question, 1 to 5 points can be assigned. An average of 3 points per question is considered “normal”. A cumulative IQCODE of ≥3.5 is regarded as “cognitive decline” [19–21].

The burden of co-morbidity was assessed using the co-morbidity and polypharmacy score (CPS) [22]. The CPS calculates the total number of chronic diagnoses and drugs taken. Standard ICU procedures were also documented.

In addition, limitations of therapy, such as withholding or withdrawing treatment, were recorded. Withholding life-sustaining therapy (e.g. mechanical ventilation, renal replacement therapy, cardiopulmonary resuscitation) was defined as not performing a measure that was indicated; withdrawing was defined as stopping any kind of life-sustaining therapy. All these decisions were at the discretion of the treating physicians and documented according to international recommendations. VIP2 recorded the exact date of treatment limitation, but VIP1 did not give specific details. Thus, the present analysis used withholding or withdrawing treatment as binary information at any time during the ICU-stay.

### Statistical analysis

Post-hoc power calculations using the 7110 octogenarians and 790 nonagenarians, primary outcome event rates of 40% versus 45%, and an alpha of 0.05, the power of the study to detect differences in 30-day mortality is 77%. Continuous data points are expressed as median ± interquartile range. Differences between independent groups were calculated using the Mann Whitney U-test. Categorical data are expressed as numbers (percentage). The chi-square test was applied to calculate differences between groups. Sensitivity analysis, analysing only patients with SOFA scores below the 75th percentile SOFA score of 10 (i.e. all patients with SOFA < 10) was performed. Univariable and multivariable logistic regression analysis was performed to assess associations with treatment limitations and mortality. Odds ratios (OR) and adjusted odds ratios (aOR) with respective 95% confidence intervals (CI) were calculated. Two sequential random effects, multilevel logistic regression models were used to evaluate the impact of being a nonagenarian on ICU- and 30-days- mortality. All patients with valid data on ICU-mortality were included. First, a baseline model with being nonagenarian as a fixed effect and ICU as random effect (model-1) was fitted. Second, to model-1, patient characteristics (SOFA, CFS, sex) (model-2) were added to the model. Adjusted odds ratios (aOR) with respective 95% confidence intervals (CI) were calculated. Sensitivity analysis, analysing only patients with and without any treatment limitation was performed. All tests were two-sided, and a *p*-value of < 0.05 was considered statistically significant. SPSS version 23.0 (IBM, USA) and MedCalc Statistical Software version 19.1.3 (MedCalc Software bv, Ostend, Belgium; https://www.medcalc.org; 2019) were used for all statistical analyses.

## Results

### Study population

This study included 7900 patients. 10% of the patients were nonagenarians. Table 1 displays the baseline characteristics of nonagenarians versus octogenarians. Nonagenarians were predominantly female (57% versus 46%, *p* < 0.001), evidenced higher rates of frailty (58% vs 42%; *p* < 0.001), disability (44% vs. 26%; *p* < 0.001) and cognitive decline (50% vs. 31%; *p* < 0.001) but lower SOFA scores at admission (6 + 5 vs. 7 + 6; *p* < 0.001). Specific ICU-treatment strategies were used, with octogenarians receiving higher rates of organ support (renal replacement therapy, mechanical ventilation, vasoactive drugs), while for nonagenarians there were higher rates of treatment limitation (40% vs. 33%; *p* < 0.001; Table 1). After discharge from the ICU, most patients had a treatment limitation; 1053 octogenarians (55% of all octogenarians leaving the ICU alive) and 182 (85%) nonagenarians left the ICU with treatment limitations in place.

**Table 1:** Baseline characteristics in the total cohort, nonagenarians versus octogenarians

	nonagenarians	octogenarians	*p*-value
	*n* = 790	*n* = 7110	
Male sex n (%)	339 (43%)	3812 (54%)	< 0.001
Age			
median (±IQR)	91 (90–93)	83 (81–86)	< 0.001
Frailty Score - CFS			
median (±IQR)	5 (4–6)	4 (3–6)	< 0.001
Frailty (CFS > 4) n (%)	454 (58)	2962 (42)	< 0.001
ADL			
median (±IQR)	5 (3–6)	6 (4–6)	< 0.001
Disability (ADL ≤4)	151 (44)	805 (26)	< 0.001
IQCODE			
median (±IQR)	3.5 (3–4)	3.2 (3–4)	< 0.001
Cognitive Decline (IQCODE ≥3.5)	149 (50)	812 (31)	< 0.001
SOFA score	6 (4–9)	7 (4–10)	< 0.001
median (±IQR)			
ICU length of stay (hours)			
median (±IQR)	84 (24–117)	54 (37–186)	< 0.001
Treatment withdrawn and/or withheld (%)	312 (40)	2302 (33)	< 0.001
NIV n (%)	168 (21)	1794 (23)	0.03
Intubation n (%)	324 (41)	3685 (52)	< 0.001
Renal replacement therapy n (%)	33 (4)	816 (12)	< 0.001
Vasoactive drugs n (%)	414 (52)	4179 (59)	0.002
Admission diagnosis n (%)			< 0.001
Respiratory failure	155 (20)	1745 (23)	
Circulatory failure	136 (17)	968 (14)	
Combined circulatory & respiratory failure	104 (13)	825 (12)	
Sepsis	74 (9)	966 (14)	
Multitrauma w/o Head Injury	23 (3)	128 (2)	
Trauma with Head Injury	18 (2)	124 (2)	
Head Injury	29 (4)	166 (2)	
Intoxication	1 (< 1)	36 (< 1)	
Cerebral Injury (Non-Traumatic)	38 (5)	469 (7)	
Emergency Surgery	91 (12)	817 (12)	
Other	91 (12)	866 (12)	

*CFS* Clinical Frailty Scale, *SOFA* Sequential Organ Failure Assessment, *ADL* Activity of Daily Life measured with the Katz index, *IQCODE* Informant Questionnaire on COgnitive Decline in the Elderly, *ICU* Intensive Care Unit, *NIV* Non-Invasive Ventilation, *SD* Standard Deviation

### Survival analysis in the total cohort

**Fig. 1:**
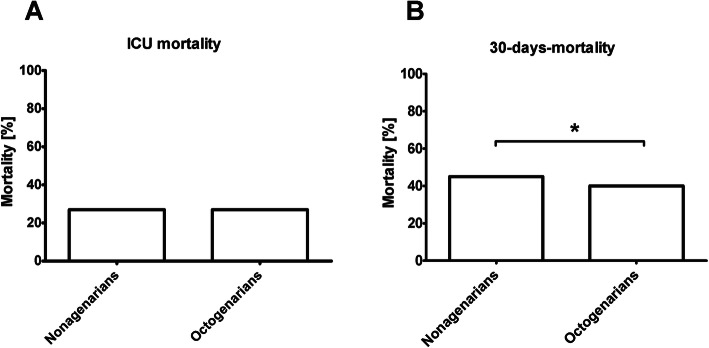
Comparison of 30-day and ICU-mortality. **A**: ICU-mortality [%], **B**: 30-day-mortality [%]. * = *p* < 0.05

The overall ICU mortality was 27% (*N* = 2134 of 7900 patients), the 30-day-mortality was 39% (*N* = 3080 of 7555 patients). Compared to the octogenarians the nonagenarians had a similar ICU mortality (27% vs. 27%; *p* = 0.973), but a higher 30-day-mortality (45% vs. 40%; *p* = 0.029, Fig. 1). Nonagenarians showed a significantly longer length of ICU-stay (84 h versus 54 h, *p* < 0.001).

### Comparison of nonagenarians versus octogenarians in the multilevel logistic regression models

After the adjustment for the ICU cluster as a random effect (model-1), nonagenarians had an increased risk for withholding life-sustaining therapy (aOR 1.54 (95% CI 1.22–1.94; p = < 0.001)), but not for withdrawal (aOR 1.03 (95% CI 0.77–1.39; p = 0,82)). Nonagenarians received significantly less mechanical ventilation, renal replacement therapy and vasoactive drugs. There was no difference between both age groups regarding the use of mechanical ventilation, vasopressors, and ICU-mortality, but an increased risk for 30-day-mortality (aOR 1.39 (95% CI 1.13–1.72); *p* = 0.002). After adding patient-specific confounders (model-2), nonagenarians demonstrated no significant risks compared to octogenarians (Table 2)

**Table 2:** Associations of primary exposure (being nonagenarian) with mortality and management strategies in a multilevel logistic regression model

	octogenarians	nonagenarians	*p*-value	model-1	model-2
Treatment withheld	27% (1945)	35% (279)	< 0.001	aOR 1.54 (95% CI 1.22–1.94; *p* = < 0.001)	aOR 0.95 (95% CI 0.67–1.36; *p* = 0.79)
Treatment withdrawn	14% (1026)	13% (102)	0.24	aOR 1.03 (95% CI 0.77–1.39; *p* = 0.82)	aOR 0.73 (95% CI 0.48–1.10; *p* = 0.13)
NIV	25% (1794)	21% (168)	0.014	aOR 0.79 (95% CI 0.61–1.03; *p* = 0.08)	aOR 0.85 (95% CI 0.59–1.22; *p* = 0.36)
Mechanical Ventilation	52% (3685)	41% (324)	< 0.001	aOR 0.72 (95% CI 0.56–0.93; *p* = 0.01)	aOR 1.26 (95% CI 0.85–1.87; *p* = 0.26)
RRT	11% (816)	4% (33)	< 0.001	aOR 0.32 (95% CI 0.19–0.53; *p* = < 0.001)	aOR 0.55 (95% CI 0.28–1.08; *p* = 0.08)
Vasoactive drugs	59% (4179)	52% (414)	< 0.001	aOR 0.74 (95% CI 0.58–0.95; *p* = 0.017)	aOR 0.90 (95% CI 0.60–1.35; *p* = 0.62)
30d Mortality	40% (2743)	44% (337)	0.029	aOR 1.39 (95% CI 1.13–1.72); *p* = 0.002)	aOR 1.25 (95% CI 0.90–1.74; *p* = 0.19)
ICU-Mortality	27% (1921)	27% (213)	0.97	aOR 1.10 (95% CI 0.87–1.40); *p* = 0.43)	aOR 0.91 (95% CI 0.63–1.32; *p* = 0.63)

*NIV* Non-Invasive Ventilation, *RRT* renal replacement therapy, *ICU* Intensive Care Unit, *aOR* Adjusted Odds Ratio, 95% CI 95% Confidence Interval

Model 1 - ICU cluster (the patient’s individual ICU) as random effect

Model 2 - Model 1 plus patient level (SOFA, CFS, age, sex)

## Discussion

This study examines the largest multi-centre prospectively recruited group of intensive care patients of 90 years and older published to date. Nonagenarians differ in their baseline risk distribution, management, and clinical outcomes from octogenarians. Nonagenarians had higher rates of frailty, cognitive impairment, and disability. However, when compared with octogenarians, nonagenarians had a lower illness severity and required less organ support. After adjustment for relevant confounders, the 30-day mortality did not differ between both groups.

Our results are in line with other studies looking at older ICU patients: Fuchs et al. evaluated a cohort of more than 7000 surgical and medical ICU patients and found age, especially above 75 years, to be an independent risk factor for mortality [9, 23]. In a large retrospective analysis of 1,807,531 patients admitted to an ICU between 1997 and 2016, Jones et al. reported increased mortality in patients older than 84 years, although they had a similar illness severity at ICU admission compared to younger patients [23]. Conversely, in a study evaluating 5882 patients after cardiac arrest, age alone was only a weak predictor of mortality [24]. In a recent study by Roedl et al., a survival rate of 46% with a good neurological outcome was reported for nonagenarians after cardiac arrest [11]. Recently, Druwé et al. performed a subgroup analysis on out-of-hospital cardiac arrests with a special interest in the resuscitation attempts in octogenarians: Most physicians considered cardiopulmonary resuscitation to be appropriate even in older patients with poor outcome perspectives [25]. Furthermore, in another study by Becker et al., the ICU mortality of nonagenarians was low at 30% and, importantly, the one-year survival was 50%, indicating outcomes “better than expected” in nonagenarians [26]. Of note, the study by Becker et al. was a single-centre study, and the number of patients who received vasoactive drugs was lower when compared to the patients in our multi-centre study. Therefore, we propose the higher mortality rates reported in the present study may be more representative of a “real-world scenario”.

Demoule et al. performed a matched case-control study in 36 nonagenarians admitted to an ICU. They were matched according to sex with 72 controls: ICU admissions chosen from the 20- to 69-year age range. They found no differences in the reason for admission, but nonagenarians suffered significantly less from pre-existing co-morbidities. Advanced life-support interventions were used equally. ICU and intra-hospital mortality, as well as the length of stay, did not differ significantly between nonagenarians and the control group [27]. Despite differences in the absolute length of stay, the trend of a shorter length of stay for older (nonagenarian) intensive care patients is consistent with previous studies [28].

Interestingly, being a nonagenarian was independently associated with the decision for withholding life-sustaining therapy, but not for withdrawing it. After adjustment for patient characteristics, nonagenarians evidenced no particular risk for treatment limitations compared to octogenarians. These findings contradict the usual expectation that physicians in general tend to be more reluctant to provide organ support to nonagenarians compared to similarly sick octogenarians. In nonagenarians, ICU re-triage should be emphasised: after an initial intensive care treatment for up to 48 h, patients should be critically evaluated in cooperation with their family and/ or carers and discharged to a normal ward for best-supportive care if further intensive care seems unethical, unjustified, or unlikely to improve outcomes. However, modern intensive care medicine is not limited to life-sustaining measures. Even beyond invasive ventilation, renal replacement therapy or cardiopulmonary resuscitation, intensive care medicine can provide valuable treatment for the patient, which might be intensified palliative therapy. Based on our data, being a nonagenarian does not represent a particular risk factor for adverse outcomes. Application of ICU re-triage could help to reduce the economic burden of ICU care in very old patients, in addition to unethical intensive care and distress caused to health care providers.

Mortality was similar between octogenarians and nonagenarians at ICU discharge and after 30 days. The long-term outcomes of the VIP2 study are awaited and will answer the question of whether this effect remains stable further over time.

An important limitation is, that we have no information about pre-ICU triage decisions, although this might be an important factor for the differences in disease illness scores and frailty between nonagenarians and octogenarians. Furthermore, this study only provides detailed information up to ICU-discharge and there was a significant rise in mortality during the 30 days after ICU-discharge, but we do not have detailed data on decisions made and developments during this period. Another limitation is that no a priori sample size calculation was made to detect a difference in the mortality between nonagenarians and octogenarians. Our post-hoc power calculation shows that the present study is likely underpowered for the primary outcome, and thus the reporting results that are at a higher risk of false positive results. However, this was counterbalanced by using a multilevel model to adjust for relevant confounders.

## Conclusion

Nonagenarian ICU patients demonstrated higher rates of frailty but had less acute organ dysfunction than octogenarians. After adjustment for multiple relevant confounders, nonagenarians did not suffer from worse outcomes compared to octogenarian ICU patients. Rather than being a nonagenarian, the severity of illness, functional capacity – and of course the patients’ will - should guide triage decisions.

## Data Availability

The anonymised data can be requested from the authors if required. The datasets analysed during the current study are not publicly available due to the different local institutional and/or licensing committees but are available from the corresponding author on reasonable request.
